# Neuroprotective properties of *Melissa officinalis* after hypoxic-ischemic injury both *in vitro* and *in vivo*

**DOI:** 10.1186/2008-2231-20-42

**Published:** 2012-10-03

**Authors:** Mohammad Bayat, Abolfazl Azami Tameh, Mohammad Hossein Ghahremani, Mohammad Akbari, Shahram Ejtemaei Mehr, Mahnaz Khanavi, Gholamreza Hassanzadeh

**Affiliations:** 1 Department of Anatomy, School of Medicine, Tehran University of Medical Sciences, Tehran, Iran; 2 Anatomical Sciences Research Center, Kashan University of Medical Sciences, Kashan, Iran; 3 Department of Toxicology - Pharmacology, School of Pharmacy, Tehran University of Medical Sciences, Tehran, Iran; 4 Department of Pharmacology, School of Medicine, Tehran University of Medical Sciences, Tehran, Iran; 5 Department of Pharmacognosy, School of Pharmacy, Tehran University of Medical Sciences, Tehran, Iran; 6 Department of Neuroscience, School of Advanced Medical Technology, Tehran University of Medical Sciences, Tehran, Iran

**Keywords:** *Melissa officinalis*, Ischemia, Cell death, Hippocampus, Neuron

## Abstract

**Background:**

Brain ischemia initiates several metabolic events leading to neuronal death. These events mediate large amount of damage that arises after some neurodegenerative disorders as well as transient brain ischemia. *Melissa officinalis* is considered as a helpful herbal plant in the prevention of various neurological diseases like Alzheimer that is related with oxidative stress.

**Methods:**

We examined the effect of *Melissa officinalis* on hypoxia induced neuronal death in a cortical neuronal culture system as *in vitro* model and transient hippocampal ischemia as *in vivo* model. Transient hippocampal ischemia was induced in male rats by tow vessel-occlusion for 20 min. After reperfusion, the histopathological changes and the levels inflammation, oxidative stress status, and caspase-3 activity in hippocampus were measured.

**Results:**

Cytotoxicity assays showed a significant protection of a 10 μg/ml dose of Melissa against hypoxia in cultured neurons which was confirmed by a conventional staining (P<0.05). Melissa treatment decrease caspase3 activity (P<0.05) and TUNEL-positive cells significantly (P<0.01). Melissa oil has also inhibited malon dialdehyde level and attenuated decrease of Antioxidant Capacity in the hippocampus. Pro-inflammatory cytokines TNF-α, IL-1β and HIF-1α mRNA levels were highly increased after ischemia and treatment with Melissa significantly suppressed HIF-1α gene expression (P<0.05).

**Discussion:**

Results showed that *Melissa officinalis* could be considered as a protective agent in various neurological diseases associated with ischemic brain injury.

## Background

Ischemic brain injury often causes irreversible neural damage. The cascade of events leading to neuronal injury and death in ischemia includes excitotoxicity, inflammation, edema, apoptosis, and necrosis [1]. In humans and experimental animals subjected to ischemia, selective and delayed neuronal death occurs in pyramidal neurons of the hippocampal CA1 region [2]. Several studies have indicated that early and late neuronal death occurring in the neurons of cortex and hippocampus after ischemia could be both apoptotic and necrotic cell death [3]. A special kind of cell death occurs some days after the initial ischemic insult, a phenomenon termed delayed neuronal death (DND) [4].

It is demonstrated that hypoxia-inducible factor-1α (HIF-1α), interleukin-1 β (IL-1β) and tumor necrosis factor α (TNF-α) expression increase in the rat brain during cerebral ischemia induced by different models of ischemia. In ischemic neuronal damage, inflammatory responses involving cytokines, adhesion molecules and leukocytes, are critical to the pathogenesis of tissue damage [5]. Local inflammatory responses contribute to secondary injury to potentially viable tissues could lead to clinical outcome in patients with ischemic stroke [6].

Reactive oxygen species (ROS) are a class of highly reactive molecules derived from oxygen and generated by some normal metabolic processes [7]. Enhanced production of ROS and the subsequent oxidative stress have been thought to play a pivotal role in ischemia/reperfusion induced neuronal death.

Natural antioxidants in plants are well known to protect human against free radicals and prevent from some diseases. *Melissa officinalis* or Lemon balm, an herb from the Labiatae family has traditionally been used for its effects on nervous system. *Melissa officinalis* leaves contain polyphenoliccompounds, such as rosmaric acid, trimeric compounds and some flavonoids [8] that can scavenge free radicals and have antioxidant properties [9]. This may prevent apoptosis induced by oxidative stress.

Essential oils derived from herbs have strong antioxidant activity due to their high contents of phenolic compounds and tocopherols [10]. Balm oil anti-diabetic and antioxidant activity reported earlier [11]. It was reported that some component of essential oil obtained from Melissia officinalis such as monoterpene aldehydes, ketones (neral/geranial, citronellal, isomenthone, and menthone) and mono- and sesquiterpene hydrocarbons (E-caryophyllene) poses free radical scavengering properties [10].

Neuroprotective effect of this plant was investigated earlier by using an *in vitro* cellular model with PC12 cell line, which was a hydrogen peroxide induced toxicity system [7]. We have reported earlier that aqueous extract of Melissa can provide neuroprotection against ecstasy induced neurotoxicity in hippocampal primary culture [12]. Recently it has been reported that oral administration of *Melissa officinalis* can increase cell proliferation and differentiation by decreasing serum corticosterone levels as well as by increasing GABA levels in the mouse dentate gyrus [13]. Infusion of lemon balm (*Melissa officinalis*) leaf for 30 days in radiology staffs exposed to low-dose ionizing radiation (x-ray) can improve oxidative stress condition and DNA damage [9].

Although several reports have been published on *Melissa officinalis*, there is no reported information, to our knowledge, regarding the *in vivo* neuroprotection properties of this plant. Studies on neurological and neuroprotective properties of *Melissa officinalis* may demonstrate the effects of this plant on the central nervous system as well as to elucidate the mechanisms involved in the activity.

The present study was carried out to examine the protective effect of Melissa in an *in vitro* hypoxia model and also the protective ability of administration before and after ischemia followed by reperfusion in hippocampal neurons as an *in vivo* model.

## Methods

### Cell culturing and treatment

Primary neuronal cultures were prepared from gestation day 15 ⁄ 16 mouse embryos (Balb c) and cultured as described previously [14]. All procedures were performed in accordance with local institutional guidelines for animal care and use.

Our procedure typically yields cultures that contain > 90% neurons and < 10% supporting cells. Neuronal purity was assessed by incubation with rabbit anti-MAP2 polyclonal antibody (Abcam,1:300 dilution) overnight at 4°C, followed by FITC-labeled goat anti-rabbit antibody (Abcam, 1:1000 dilution) for 1 h at room temperature, Hoechst 33342 counterstaining (1:10000 dilution) for 10 minutes, and cover slipping in Mowiol mounting media (Sigma, Germany).

Cultures were maintained at 37°C in a humidified atmosphere containing 95% air–5% CO2 for 7 days. Prior to experiments, the medium was replaced by supplemented neurobasal medium after 24 h and changed every 3 days after.

### Treatment with melissa and hypoxia

Balm Oil (B4008 Sigma, Germany) was serially diluted in serum free medium. Cultures were pretreated with Melissa for 2 h in normal incubator (95% air i.e., ~21% O2 –5% CO2 equilibrated to 37°C and 95% humidity) as normoxia incubator before their transfer in to hypoxia incubator (90% N2–5% CO2 and 5% O2 equilibrated to 37°C and 95% humidity) for 24 h [15]. After 24 h of hypoxia, cultures were removed from the hypoxic chamber, and were returned to normoxia incubator for another 4 h reperfusion period until analysis.

### MTS/LDH assay

Cell membrane integrity was determined by lactate dehydrogenase (LDH) using CytoTox-ONE^TM^ Homogeneous Membrane Integrity Assay according to the manufacturer’s instructions (Promega, Germany). Two vials per experiment were treated 2 h with 2μl lysis solution containing Triton X-100 as positive control. Having no background from mediums containing Melissa was assured by reading their absorption with 492nm - 620nm filters which were near zero (Data not shown).

Cell viability was determined by MTS using One Solution Cell Proliferation Assay according to the manufacturer’s instructions (Promega, Germany).

### Propidium Iodide (PI) /Hoechst staining and fixation

Cell death was determined by 4 h incubation of cultures in medium containing 4 μl/ml PI (500μg/ml) (Sigma, Germany) before fixation. Viable neurons with sufficient cell membrane integration could pump PI out hence late apoptotic and necrotic cells could not do that and in this experiment are presented as PI positive neurons. Cells were then fixed with 4% formaldehyde for 15 minutes. Staining was done in darkness to prevent bleaching. A repeat count of necrotic cells was performed, the cells were kept at 4°C in PBS overnight, and then necrotic cells were again counted. This provided an index of the preservation of the PI stain after fixation. Hoechst 33342 0.1 μg/ml (Sigma, Germany) staining was done for 10 min after fixation in order to normalize PI positive neurons to the total number of nuclei in the field which were stained with Hoechst. Cells were visualized using an Axioskop 2 plus microscope (Carl Zeiss, Germany) with a 40× phase contrast water immersion objective, and images captured using an AxioCam HRc camera controlled by AxioVision software.

### Preparation of *in vivo* ischemia model and drug administration

All *in vivo* experiments were performed on male Sprague–Dawley (250 to 280 g) rats. Before the induction of transient cerebral ischemia, rats were anaesthetized with chloral-hydrate (350 mg/kg, i.p.) and the body temperature was maintained at 37±0.5°C throughout the procedure with the use of a heating pad. A midline incision was made on the ventral side of the neck to expose the common carotid arteries. The common carotid arteries were isolated from vagus nerves and clamped with non-traumatic aneurysm clips [16]. Sham-operated control rats underwent the same procedure, but without common carotid artery occlusion. The surgery was accompanied by a 100% survival rate following common carotid occlusion. Carotid artery blood flow was reperfused by releasing the clips following 20 minutes occlusion. The average duration of operation was about 30 minutes.

Plant material diluted with physiological saline to obtain a final concentration of 10%. To find out the most effective dose of M. officinalis we used several dosage (50, 100, 200 and 400 mg/kg) of plant material [13]. In order to dose response data, 100 mg/kg of plant material was selected (Data not shown). 100 mg/kg Melissa was gavaged orally using the gavage needle every day for two weeks before operation as pretreatment and also continued after ischemia in different reperfusion time points. Animal groups were three: sham-operated group also considered as control, vehicle-treated group and Melissa treated group.

### Caspase-3 assay

To recognize the *in vivo* neuroprotective properties of *Melissa officinalis*, we examined caspase-3-like activity at different time periods after ischemia. Fluorometric assay kit for caspase-3 activity (BD Pharmingen^TM^) was used according to manufacturer’s instruction. Rats were killed, brains were removed and both hippocampi were rapidly dissected out on ice, were minced with scissors, and homogenized in ice-cold lysis buffer containing 10 mM Tris–HCl; 10 mM NaH2PO4/NaHPO4 (pH 7.5); 130 mM NaCl; 1% TritonR-X-100; 10 mM NaPPi (sodium pyrophosphate). Lysates were centrifuged (14000 rpm, 10 min, 4°C) and the supernatants were taken and kept in −80°C for further use. Protein concentration was measured using Bradford protein assay [17]. 100 μg total protein was incubated for 1 h at 37°C with reaction buffer [40 mM HEPES (pH 7.5); 20% glycerol; 4 mM DTT] and the fluorogenic substrate Ac-DEVD-AMC [N-acetyl-Asp-Glu-Val-Asp-AMC (7-amino-4-methylcoumarin)]. The amount of 7-amino-4-methylcoumarin liberated from the Ac-DEVD-AMC fluorogenic peptide via the action of caspase-3 was measured on a spectrofluorometer with an excitation wavelength of 380 nm and an emission wavelength of 420 nm.

### In situ labeling of DNA fragmentation

TUNEL methodology was used to assess neuronal cell death in the CA1 (Cornu Ammonis) region of the hippocampus. Hence it takes 2–3 days for the neuronal damage of CA1 to become morphologically obvious, cresyl violet and TUNEL staining were done at day 5 after reperfusion. Apoptosis occurring *in vivo* was assessed by TUNEL labeling [18]. An in situ cell death detection kit (Roche, Germany) was used to carry out TUNEL staining on sections according to the manufacturer's instructions. Staining was visualized with diaminobenzidine. Each group contained 7 animals and from each animal 3 sections stained. The number of surviving neurons and TUNEL-positive cells per millimeter linear length in the CA1 region [18,19] of the dorsal hippocampus was counted by an investigator who was blinded to the experimental conditions.

### RT-PCR

Expression of HIF- α, TNF-α and IL1-β are increased after permanent or transient cerebral ischemia [20-23]. Although anti-inflammatory strategies to attenuate ischemic brain injury have been inadequate, we carried out to examine the gene expression of HIF-1α, IL-1β and TNF-α after ischemia and anti-inflammatory properties of *Melissa officinalis*.

After 20 minutes ischemia and two days reperfusion, animals were sacrificed by decapitation, brains were removed rapidly, and hippocampi were dissected quickly, placed in RNA later RNA Stabilization Reagent (Qiagen, Germany) over night at 4°C and finally stored at −80°C until used. Total RNA was extracted using the RNeasy Mini Spin Columns Collection Tubes (Qiagen, Germany). Reverse transcription was done using the RT-PCR technology according to manual instruction (Bioneer, South Korea). Using specific primer sets (Table 1), aliquots of cDNA were amplified by a PCR machine (Peqlab, Germany), with initial denaturation at 94°C for 5 min, followed by 30 cycles of denaturation at 94°C for 30 s, annealing at variable primer-specific temperatures for 30 s, 45 s for extension at 72°C, and a further 5 min final extension at 72°C on completion of the cycles. Cycle optimization was performed for each primer set before PCR. The amplified products were subjected to 1% (W/V) agarose gel for electrophoresis, stained with ethidium bromide, then observed and photographed under an ultraviolet lamp in a gel imaging system. PCR product bands were analyzed with the ImageJ 1.440 software (National Health Institute, USA), ratios of each target gene to that of the house-keeping gene GAPDH (glyceraldehyde-3-phosphate dehydrogenase) was taken as the semiquantitative results of the samples.

**Table 1:** Primers used for RT-PCR, F, forward sequence; R, reverse sequence, and primer source

**Gene**	**Annealing temperature (°C)**	**Product size (bp)**	**Sequence (5’–3’)**	**accession number**
HIF-1α	61	197	F TCAAGTCAGCAACGTGGAAG	[GenenBank:024359.1]
			R TATCGAGGCTGTGTCGACTG	
IL1-b	60	209	F CTGTGACTCGTGGGATGATG	[GenenBank:031512.2]
			R GGGATTTTGTCGTTGCTTGT	
TNF-α	60	209	F CTCCCAGAAAAGCAAGCAAC	[GenenBank:012675.3]
			R CGAGCAGGAATGAGAAGAGG	
GAPDH	59	161	F CATCACCATCTTCCAGGAGCGAGA	[GenenBank:017008.3]
			R CAGCGGAAGGGGCGGAGA	

### Determination of Trolox Equivalent Antioxidant Capacity (TEAC)

Oxidative damage in the ischemic animals was measured by the level of antioxidant capacity in the tissue homogenates. Tissue homogenates TEAC was determined by its ability to inhibition of peroxidase-mediated formation of the 2,2'-azino-bis-3-ethylbenzthiozoline-6-sulfonate (ABTS^.+^) radical [24]. 50 μL of samples were loaded onto respective wells on the 96-well microplate. 200 μL Chromagen (ABTS) (Sigma-Aldrich Inc., USA) was then added to these wells and the mixture left to react at 25°C for 6 minutes before reading the absorbance at 750 nm using the Bio-Rad Benchmark Plus Microplate Reader. The capacity of the homogenate antioxidant to inhibit ABTS oxidation was compared to the water-soluble vitamin E analogue (trolox) (Sigma-Aldrich Inc., USA). The TEAC values were determined from the trolox standard curve. Results were expressed as millimoles per trolox equivalents per liter of homogenate. TEAC values were taken as the total antioxidant capacity in the tissue homogenates samples of the animals.

### Assay for thiobarbituric acid reactive substances (TBARS)

Oxidative damage in the ischemic animals was measured by the level of malondialdehyde (MDA) formed in the tissue homogenates. MDA levels in hippocampal tissue were determined according to the method of Ohkawa et al. [25]. Briefly, the hippocampus was homogenized in cold 0.1M phosphate buffer (pH 7.4) to make a 10% homogenate. Then homogenates were centrifuged for 30 min at 3000×g at 4°C. An aliquot of supernatant was added to a reaction mixture containing 100μl of 8.1% sodium dodecyl sulphate, 750μl of 20% acetic acid (pH 3.5), 750μl of 0.8% thiobarbituric acid and 300μl distilled water. Samples were then boiled for 1 h at 95°C and centrifuged at 4000×g for 10 min. The absorbance of the supernatant was measured spectrophotometrically at 532nm and results were expressed as nanomoles of MDA per mg of protein.

### Statistical analysis

The values are expressed as mean ± standard deviation (SD). The results were computed statistically (Graphpad Prism 5.0) using *t* test.

Degrees of significance were assessed by three different rating values: P<0.05 = *(significant), P<0.01 = ** (highly significant), and P<0.001 = *** (extremely significant). For clarity, data in figures are expressed relative to their respective controls.

## Results

### Cortical neuronal viability after hypoxia

**Figure 1:**
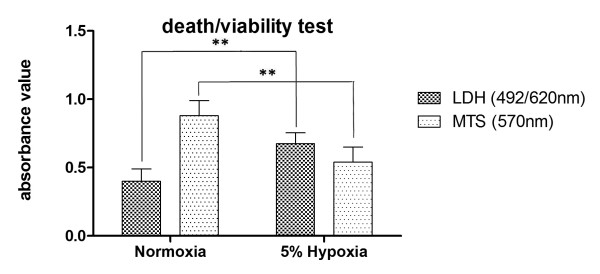
**MTS/LDH release in normoxic and hypoxic conditions.** Hypoxia induced by 5% oxygen for 24h and followed by 4h reperfusion in normal condition. Results are given in absorbance values to control groups which were all the time in normoxia condition. LDH release is increased and MTS activity is decreased significantly in hypoxic condition. **P< 0.01, vs. normoxia.

Almost all types of CNS cells appear to be vulnerable to hypoxia, including astrocytes, microglia and neurons, although regional and cellular differences with respect to exposure time may also be considerable. In a first step, we aimed to analyze the vulnerability of cortical neurons to a given hypoxia percent and exposure duration. Cell viability was assessed by lactate dehydrogenase (LDH) release in the culture supernatant and metabolic activity of cells by 3-(4,5-dimethylthiazol-2-yl)-5-(3-carboxy methoxyphenyl)-2-(4-sulfophenyl)-2H-tetrazolium) (MTS) in adherent neurons. As shown in Figure 1 hypoxia induced a significant increasein LDH activity in the supernatant. This was paralleled by a decrease in corresponding metabolic activity by MTS assay. After 24 h of 5% hypoxia administration and 4 h reperfusion the viability of cortical neurons observed to have declined by approximately 55% (P< 0.01, vs.normoxia), as indicated by a massive increase in LDH activity. Metabolic activity was also decreased around 20% (P< 0.01, vs.normoxia), as shown by MTS absorbance in Figure 1.

### Dose response of cortical neurons to M. Officinalis

**Figure 2:**
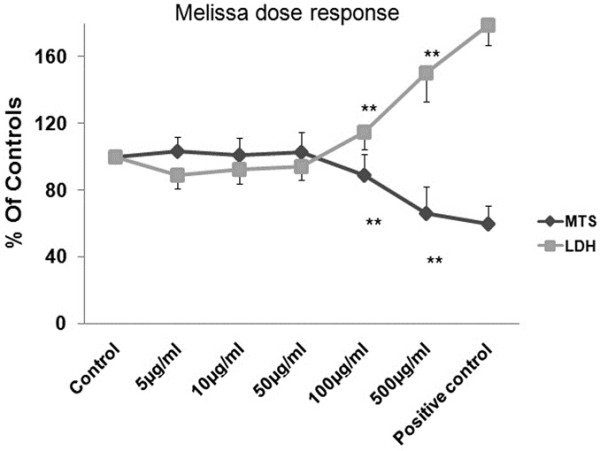
**Melissa dose response in normoxia condition.** There is no change in MTS activity or LDH release in low concentrations (5, 10 and 50 μg/ml) of Melissa in normoxic condition while higher concentrations (100 and 500 μg/ml) cause significant decrease in neuronal viability which is seen in MTS reduction and LDH increase. Results are given as a relation of both MTS and LDH values to their controls which were in normoxia condition.**P< 0.01, vs. control or normoxia.

In this step, we intended to see the response of cortical neurons to different concentrations of Melissa. For this purpose we did a set of LDH and MTS assays in normoxic condition in order to find the suitable dose of Melissa for further administration in hypoxic condition. As shown in Figure 2 there was no change in MTS activity or LDH release in low concentrations (5, 10 and 50 μg/ml) of Melissa in normoxic condition compared to control group. But higher concentrations (100 and 500 μg/ml) were seemed to be toxic and caused significant decrease in metabolic activity (P< 0.05, vs. control) (Figure 2).

### Protective effects of M. Officinalis during hypoxia *in vitro*

**Figure 3:**
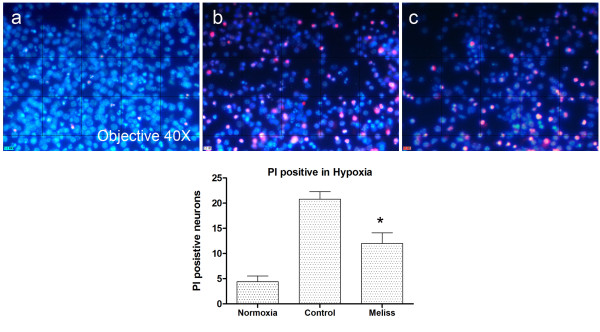
**PI positive neurons in normoxia (a), hypoxia (b) and 10 μg/ml treatment of melissa (c).** Total number of PI positive neurons is higher than hypoxic groupafter Melissa treatment (b and c). PI positive neurons (red) were counted and normalized to all cells counterstained with Hoechst (blue).*P< 0.05, vs. control.

A 24h hypoxia exposure followed by 4h reperfusion resulted in an approximately half maximal decline in cell viability; this protocol was used for all further experiments. To recognize the protective properties of Melissa on neuronal hypoxia induced death, neurons were treated with neurobasal medium containing 10 μg/ml concentration of Melissa. As shown in Figure 3, PI positive cortical neurons increased significantly after hypoxia protocol comparing to normoxia (a and b) and single dose of Melissa significantly reduced PI positive cell count comparing to cultures exposed to hypoxia without any treatment (b and c) (P< 0.05, vs. hypoxia controls).

### Caspase-3 activity assay

**Figure 4:**
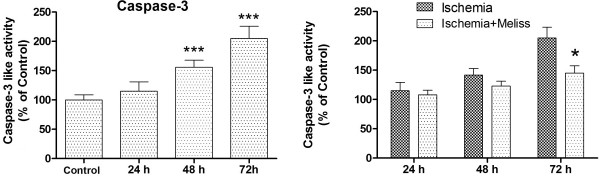
**Caspase-3-like activity.** Left: Temporal profiles of caspase-3-like activity in the hippocampus after transient ischemia. Results are given in % of control group absorbance value. Significant increase in caspase-3 activity is observed after 48h and 72h reperfusion. ***p < 0.001, vs. control animals(n=8). Right: Treatment with 100 mg/kg of *Melissa officinalis* attenuated the increased caspase-3 like protease activity. *P< 0.05, vs. ischemia group (n=11).

After 20 minutes ischemia followed byseveral time pointsof reperfusion, caspase-3like activity in the hippocampus was increased significantly (Figure 4) (P< 0.01, vs. controls animals).

At day 3 caspase-3 activity as seen in Figure 4 was at highest level. On the basis of the above mentioned result and also a recent similar study [26], we assessed the effect of *in vivo* treatment with *Melissa officinalis* on the caspase-3like protease activity at day 3of reperfusion. Treatment with 100 mg/kg of Melissa oilattenuated the increased caspase-3 like protease activity significantly (P< 0.05, vs.ischemia group) (Figure 4).

### In situ labeling of DNA fragmentation

**Figure 5:**
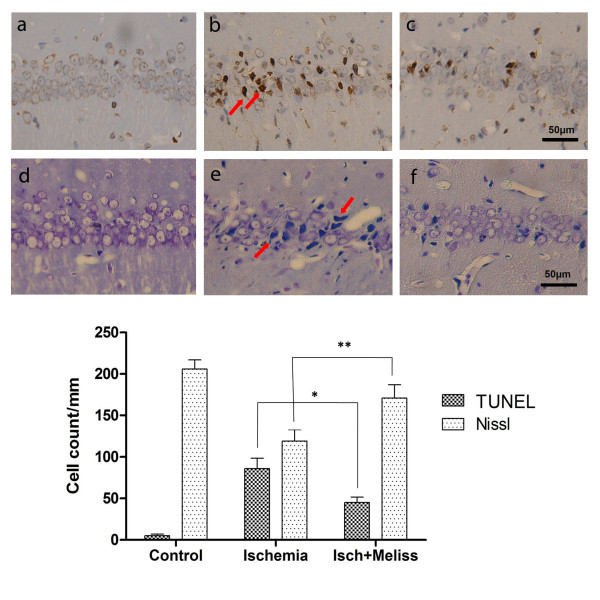
**Micrographs of TUNEL-positive cells and cresyl-violet stained cells.** Representative micrographs of TUNEL-positive cells (dark brown) (**a**–**c**) and cresyl-violet stained cells (**d**–**f**) in the hippocampal CA1 region from sham (**a** and **d**); ischemic (**b** and **e**); Melissa-treated animals (**c** and **f**) at 5 days of reperfusion. Scale bar: 50μm. Graph shows the comparison of apoptotic (TUNEL-positive) and surviving (Nissel positive) neurons between groups. *p < 0.05, **p < 0.01 compared with ischemia group (n=7).

TUNEL staining revealed that many TUNEL-positive neurons were present in the hippocampal CA1 region of ischemic rats (Figure 5b). Few TUNEL-positive cells were found in the CA1 region of sham operated rats (Figure 5a). The number of TUNEL-positive cells was reduced by treatment with plant material (Figure 5c).

Cresyl violet staining revealed extensive neuronal loss in the CA1 region of ischemic rats (Figure 5e). No cell damage was evident by the cresyl violet staining in the CA1 region of sham-operated rats (Figure 5d). Hippocampal neuronal damage was decreased by treatment with 100mg/kg Melissa administration (Figure 5f).

### RT-PCR

**Figure 6:**
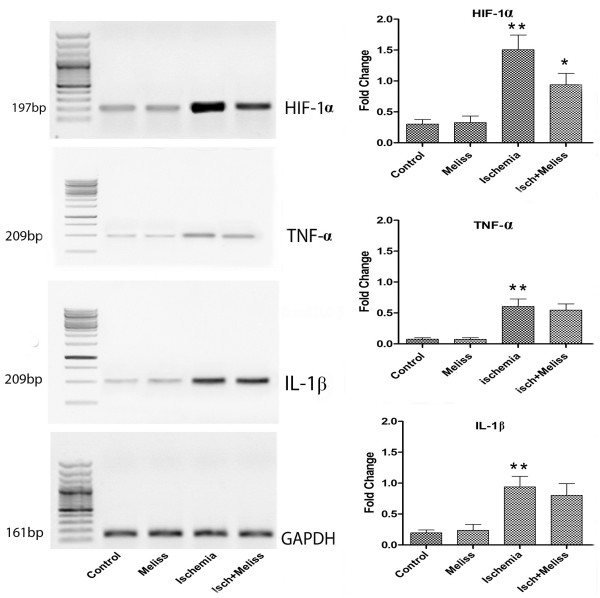
**RT-PCR analysis of HIF-1α, TNF-α and IL-1β gene expression in the hippocampus of ischemic rats after ischemia.** 100 mg/kg treatment of Melissa significantly suppressed the expression of HIF-1α in the ischemic hippocampus (P<0.05) while TNF-α and IL1-β expression have not been significantly decrease (n=8).

Expression of HIF- α, TNF-αand IL1-β are increased after permanent or transient cerebral ischemia [21-23]. We further examined the expression of HIF-1α, TNF-α and IL1-β after ischemia and when treated with Melissa in the ischemic rat brain. On the basis of similar studies [27,28] 2days after induction of ischemic brain injury, expression of HIF- α, TNF-α and IL1-β were measured. As shown in Figure 6, mRNA expressions of HIF-1α, TNF-α and IL1-β were up-regulated after ischemia/reperfusion injury (p < 0.01). Melissa (100 mg/kg) treatment suppressed the expression of HIF-1α in the ischemic hippocampus (P<0.05) while TNF-α and IL1- β expression have not been decreased significantly (Figure 6).

### Lipid peroxidation and antioxidant capacity

**Figure 7:**
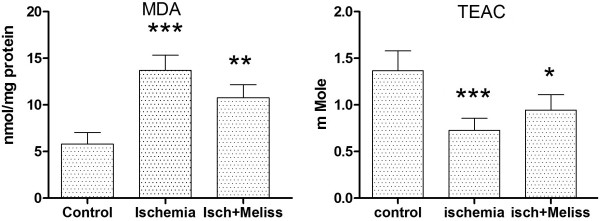
**Lipid Peroxidation and Antioxidant Capacity.** Left: Comparison of the level of MDA (left) and TEAC (right) inthe hippocampus of sham operated, ischemic and *Melissa officinalis* treated ischemic animals. Treatment with 100 mg/kg of Melissa decreased the elevated MDA levels in the hippocampus (**p < 0.01) and attenuates decrease of antioxidant status of ischemic animals (*p < 0.05) (n = 8 each).

In ischemic animals, on the basis of a recent similar study,MDA level and antioxidant status in hippocampus measured at day 2of reperfusion [18]. MDA level increased after 2 days of reperfusion. In balm oil treated (100 mg/kg) ischemic animals MDA level was significantly lower than sham operated animals (P< 0.01) (Figure 7).

The antioxidant status was assessed by studying the level of TEAC in the tissue homogenates. A lowered antioxidant defense system in ischemic animals compared to the sham operated animals is noted. At day 2 of reperfusion TEAC concentrations in tissue homogenates of ischemic animals were lower than sham operated animals. In balm oil treated (100 mg/kg) ischemic animals the level of TEAC was significantly higher than ischemic animals (p < 0.05) (Figure 7).

## Discussion

Increasing evidence has indicated that production of free radicals after cerebral ischemia and reperfusion, caused oxidative stress which is involved in ischemic brain damage [29]. During ischemia and especially reperfusion, free radicals are expected to attack lipids and proteins of the cell membrane and DNA [30]. Novel therapeutic neuroprotective strategies support the applications of ROS scavengers and induction of endogenous antioxidants, such as natural antioxidants, for example plant derived polyphenolic compounds, for the treatment of neurodegenerative diseases [18,31,32]. It is known that some compounds of *Melissa officinalis* have antioxidant activity which is due to its free Radical Scavenging Capacity (RSC) [33]. Antioxidant activity of Melissa has previously been reported in different studies [8,33-35]. In addition it is reported that this plant has protective effect on hydrogen peroxide induced toxicity in PC12 cells which have some characteristics of neurons [7], but the protective effects in primary culture of neurons after hypoxic stress have never been reported. In this study, 24 h exposure of primary cortical neurons to 5% hypoxia followed by 4 h reperfusion reduced both cell viability and metabolic activity to around 55% and 20%, respectively. This could be considered as an approval on hypoxia system. Hoechst/PI staining of neurons showed that 10 μg/ml concentration of Melissa could significantly reduce cell death. Although, analysis of dose response results showed that high doses (100–500 μg/ml) could worsen the condition with500 μg/ml dose as half maximal inhibitory concentration (IC_50_) of Melissa. These results suggest that some concentrations of Melissa have protective activities in neurons and may keep them safe from oxidative stress. *In vitro* results lead us to postulate the hypothesis that Melissa could have some protective effects on neurons in the brain. Therefore, we examined its effects in ischemic model of brain injury as *in vivo* model. Investigations showed that after induction of ischemia, Caspase-3 activity in hippocampus significantly increased, and there were many TUNEL positive neurons in CA1 area. TUNEL is a common method for detecting DNA fragmentation that results from apoptotic signalling cascades. The assay relies on the presence of nicks in the DNA which can be identified by terminal deoxynucleotidyl transferase or TdT, an enzyme that will catalyse the addition of dUTPs that are secondarily labelled with a marker. It may also label cells that have suffered severe DNA damage.

Mechanisms leading to DNA fragmentation following ischemia may not be clear but a specific DNase, caspase-activated DNase (CAD) that cleaves chromosomal DNA appears to be an important enzyme in apoptotic cell death. CAD is generally found as a complex with ICAD (inhibitor of CAD) which serves to limit its DNase activity. After initiation of apoptosis signals, caspases, in particular caspase-3, cleave ICAD to dissociate CAD from ICAD, thereby allowing CAD to cleave chromosomal DNA [36]. So we investigate both caspase-3 activity and DNA fragmentation. This could suggest that Melissa provided neuroprotection against cerebral I/R injury in the rat brain. These results were consistent with our *in vitro* study and this is the first time that shows protective activity for *Melissa officinalis* after brain ischemia.

In present study, the increased MDA and decreased antioxidant defense system in the hippocampus of the ischemic group as compared to the sham group suggests a state of enhanced oxidative stress in ischemia–reperfusion injury. Apoptotic cell death occurs in response to various stimuli including oxidative stress [37]. The brain is very vulnerable to oxidative stress due to its high polyunsaturated fatty acid (PUFA) content, which is particularly susceptible to ROS damage [38]. During ischemia, superoxide anions and hydrogen peroxide form and cannot be readily scavenged. Lipid peroxidation (LPO) is one of the important markers of oxidative damage in the ischemic cascade as PUFA constitutes a major component of brain, which results in the formation of lipid peroxides, and may affect a variety of cellular functions involving proteins namely: receptors, signal transduction mechanisms, transport systems and enzymes [39]. Treatment of ischemic rats with Melissa significantly inhibited MDA level and attenuated decrease of antioxidant capacity in the hippocampus. It is well documented that attenuating oxidative stress is important in evolving neuroprotective strategies for enhanced neuronal survival after cerebral ischemia [18]. We suggest that some components of plant material that have antioxidative properties can attenuate oxidative damage induced by ischemic brain injury.

Ischemic brain injury induced increase in HIF-1α, IL-1β and TNF-α mRNA level, this can represent the inflammatory response of neuronal or glial cells suffering from the ischemic insult. It is demonstrated that expression of HIF-1α and HIF-1β mRNA in rat and mouse has been up-regulated in ischemic brain injury [23]. In this study, showed that the elevation of active caspase-3 expression occurred as well as HIF-1α expression after ischemic injury, and these events could be significantly suppressed by treatment with Balm oil. Hypoxia can cause HIF-1α to bind with p53 in order to stabilize it, and also activates the expression of various genes, including bax. In this study, plant material can down-regulate the transcription of HIF-1α during hippocampal ischemia and inhibit caspase-3 activation. Proinflammatory and immunomodulatory cytokines like TNF-α, IL-1β and IL-6 mRNA level increases after focal ischemia [22], implicating that these cytokines can develop ischemic brain injury. Gene expression of TNF-α and IL-1β, after cerebral ischemia is up-regulated. Treatment of ischemic animals with Melissa did not effectively inhibit mRNA expression of TNF-α and IL-1β, indicating that the inhibition of TNF-α and IL-1β might not be the neuroprotective mechanism for plant materialin ischemic brain injury.

## Conclusions

In conclusion, results implicate that *Melissa officinalis* has shown protective effect on ischemic damage mediated by the inhibition of HIF-1α and oxidative stress, followed by the inhibition of apoptosis.

These results propose the potential use of *Melissa officinalis* or its constituents for central nervous system diseases and as a neuroprotective agent to prevent disorders involved with oxidative stress. Experiments are necessary to identify which of the plant components are responsible for these activities.

## Competing interest

The authors have no financial interest to declare. There is no conflict of interest to declare.

## Authors’ contributions

GH designed the study. MB, AAT, MHG, MA, SEM, MK, performed the experiments and analyses. MB and AAT wrote the paper. All authors read and approved the final manuscript.
